# DNA repair factor KAT5 prevents ischemic acute kidney injury through glomerular filtration regulation

**DOI:** 10.1016/j.isci.2021.103436

**Published:** 2021-11-14

**Authors:** Akihito Hishikawa, Kaori Hayashi, Akiko Kubo, Kazutoshi Miyashita, Akinori Hashiguchi, Kenichiro Kinouchi, Norifumi Yoshimoto, Ran Nakamichi, Riki Akashio, Erina Sugita, Tatsuhiko Azegami, Toshiaki Monkawa, Makoto Suematsu, Hiroshi Itoh

**Affiliations:** 1 Division of Nephrology, Endocrinology and Metabolism, Department of Internal Medicine, Keio University School of Medicine, 35 Shinanomachi, Shinjuku-ku, Tokyo 160-8582, Japan; 2 Department of Biochemistry, Keio University School of Medicine, Tokyo, Japan; 3 Department of Pathology, Keio University School of Medicine, Tokyo, Japan; 4 Medical Education Center, Keio University School of Medicine, Tokyo, Japan

**Keywords:** Pathophysiology, Cell biology

## Abstract

The “preconditioning effect” in AKI is a phenomenon in which an episode of ischemia-reperfusion results in tolerance to subsequent ischemia-reperfusion injury. However, its relationship between DNA damage repair has not been elucidated. Here, we show the role of KAT5 in the preconditioning effect. Preconditioning attenuated DNA damage in proximal tubular cells with elevated KAT5 expression. Ischemia-reperfusion (IR) injuries were exacerbated, and preconditioning effect vanished in proximal tubular-cell-specific KAT5 knockout mice. Investigation of tubuloglomerular feedback (TGF) by MALDI-IMS and urinary adenosine revealed that preconditioning caused attenuated TGF at least in part via KAT5. In addition, K-Cl cotransporter 3 (KCC3) expression decreased in damaged proximal tubular cells, which may be involved in accelerated TGF following IR. Furthermore, KAT5 induced KCC3 expression by maintaining chromatin accessibility and binding to the KCC3 promoter. These results suggest a novel mechanism of the preconditioning effect mediated by the promotion of DNA repair and attenuation of TGF through KAT5.

## Highlights

• KAT5-mediated DNA damage repair acts against ischemia-reperfusion (IR) injuries
• K-Cl cotransporter3 (KCC3) expression is decreased in damaged proximal tubular cells
• Decreased KCC3 may lead to AKI via acceleration of tubuloglomerular feedback
• KAT5 induces KCC3 expression through an epigenetic mechanism

## Graphical abstract

**Figure:**
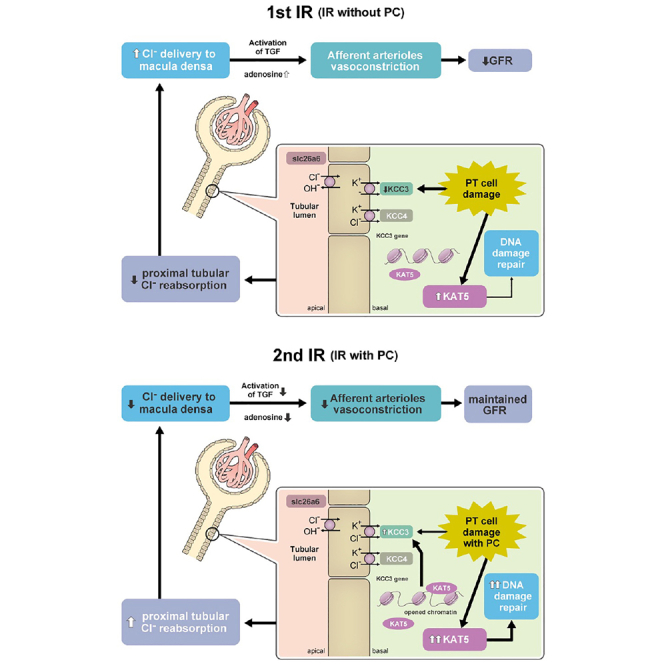

## Introduction

The concept of the “memory effect,” the persistent effects of transient treatments or insults on disease progression and complications for longer periods, has been recognized in large clinical trials of diabetes (Diabetes Control and Complications Trial/Epidemiology of Diabetes Interventions and Complications Research Group et al., 2000; Nathan et al., 2003, 2005). The existence of memory effects has also been suggested in hypertension and atherosclerosis (Itoh et al., 2018). One of the possible mechanisms of the memory effect is epigenetic changes, including altered DNA methylation (Chen et al., 2016, 2020; Kato and Natarajan, 2019). These epigenetic memories stored in the kidney were suggested in human studies (Gluck et al., 2019; Park et al., 2019) and investigated in animal models, including ours (Hayashi et al., 2014, 2015).

Acute kidney injury (AKI) is a worldwide public health problem associated with not only acute hospital mortality but also an increased risk of chronic kidney disease (CKD) and long-term mortality (Coca et al., 2009, 2012). After recovery from AKI, several memory effects have been described previously. One is the “preconditioning effect” (Kapitsinou and Haase, 2015; Wever et al., 2012; Zager, 2013), which is considered to be a renoprotective memory of the relatively short term, and the other is an increased risk of CKD after recovery of AKI, which is a detrimental memory of the chronic phase (Bonventre and Yang, 2011; Zager, 2013). The preconditioning effect is mainly reported in ischemic AKI in which an episode of ischemia-reperfusion results in tolerance to subsequent ischemia-reperfusion injury and may also feasibly prevent AKI after surgery in humans (Zarbock et al., 2015; Zuk and Bonventre, 2015). However, the precise mechanism has not been adequately elucidated.

Recently, we demonstrated the association of DNA damage repair with altered DNA methylation in kidney podocytes (Hishikawa et al., 2019). The DNA repair system is essential for maintaining genome integrity, and a recent report has suggested that it plays a central role in recovery and longevity following AKI (Kishi et al., 2019); therefore, it is of great interest whether DNA repair and associated epigenetic changes may be involved in the formation of an AKI memory, that is, the preconditioning effect.

We have recently reported that KAT5 plays a key role in DNA damage repair and is essential for maintaining healthy podocytes (Hishikawa et al., 2019). KAT5 is a histone acetyltransferase implicated in transcriptional control and DNA double-strand break repair through the acetylation of ataxia telangiectasia mutated (ATM), histone H4, and H2AX (Berkovich et al., 2007; Cann and Dellaire, 2011; Wu et al., 2007). Here, we report that KAT5 in proximal tubular (PT) cells mediates the AKI preconditioning effect through promotion of DNA repair and protection of PT cells. Furthermore, KAT5 is involved in the regulation of tubuloglomerular feedback (TGF) through epigenetic regulation of K-Cl cotransporter 3 (KCC3) in PT cells, in addition to a role in DNA DSB repair.

## Results

### Ischemic preconditioning causes an increase in KAT5 expression and attenuates DNA damage in proximal tubular cells

**Figure 1:**
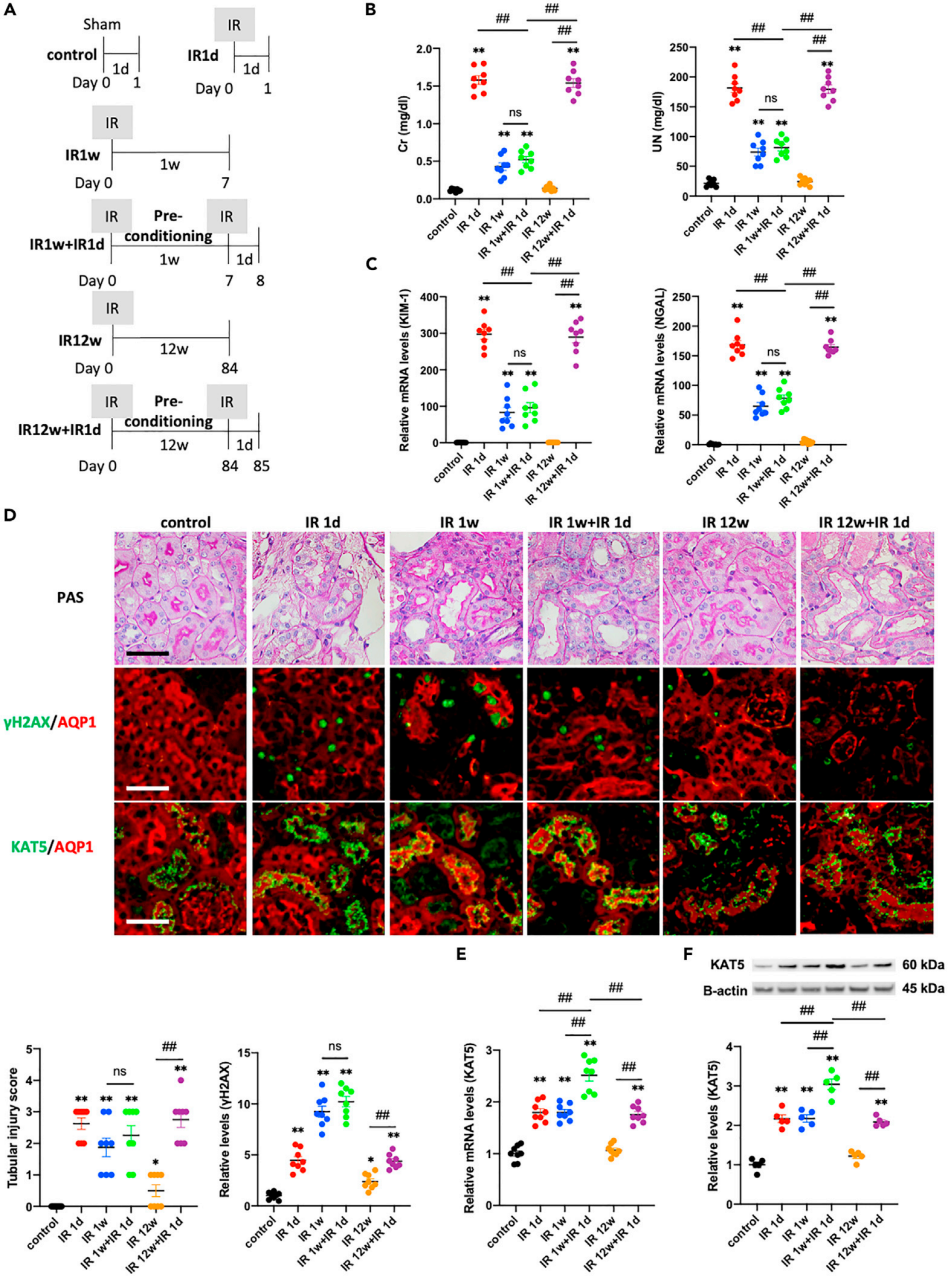
Ischemic preconditioning causes an increase in KAT5 expression and attenuates DNA damage in proximal tubular cells (A) Experimental protocol. Ischemic preconditioning was performed 1 week prior to the injury (n = 5–8 per groups). (B) Serum creatinine and UN levels. (C) Expression of KIM-1 and NGAL in the kidney cortex. (D) (Upper panels) Representative photomicrographs of PAS staining and immunofluorescence double staining of γH2AX or KAT5 (green)/AQP1 (red) in kidney cortex. Scale bar, 50 μm. (Lower panels) Tubular injury scores and quantification of the immunolabeled double-positive area. (E) Real-time RT-PCR analysis of KAT5 expression in the kidney cortex. (F) KAT5 expression in isolated proximal tubular cells. Data represent the mean ± SEM. ∗p < 0.05, ∗∗p < 0.01 versus controls, #p < 0.05, ##p < 0.01 versus the respective groups.

To examine the impact of ischemic preconditioning on renal function, an experimental IR model in mice was used by clamping the bilateral renal arteries for 30 min followed by reperfusion. Ischemic preconditioning was performed in the same manner one week prior to IR injury (Figure 1A). Elevated serum creatinine (Cr), urea nitrogen (UN), and the expression of kidney injury molecule-1 (KIM-1) and neutrophil-gelatinase-associated lipocalin (NGAL) in the kidney cortex observed at 24 h after IR injury were attenuated by ischemic preconditioning (IR1d versus IR 1w + IR 1d) (Figures 1B and 1C). The level of γH2AX, a DNA double-strand break (DSB) marker, was significantly elevated 24 h after IR injury in AQP1-positive proximal tubular cells by immunofluorescence staining (control versus IR 1d) (Figure 1D). On the other hand, DNA DSBs and tubular injury scores were not additionally increased following IR injury with preconditioning (IR 1w versus IR 1w + IR 1d). IR injury with preconditioning induced increased KAT5 expression compared with IR injury without preconditioning (IR1d versus IR 1w + IR 1d) (Figures 1D–1F). To investigate the duration of the preconditioning effect in our model, kidney function was evaluated in mice with preconditioning 12 weeks prior to IR and compared with those with preconditioning 1 week prior to IR (Figure 1A). Preconditioning 12 weeks prior to IR caused less attenuation of kidney dysfunction compared with that 1 week prior to IR injury (IR 1w + IR 1d versus IR 12w + IR 1d) (Figures 1B and 1C), as reported previously (Park et al., 2003). KAT5 expression was less increased by preconditioning 12 weeks prior to IR (IR 1w + IR 1d versus IR 12w + IR 1d) (Figures 1E and 1F). The expression of other DNA repair factors was also increased after IR with preconditioning (Figure S1). These results indicate that elevated DNA repair factors, including KAT5, may have a role in the preconditioning effect through attenuation of DNA damage in PT cells.

**Figure 2:**
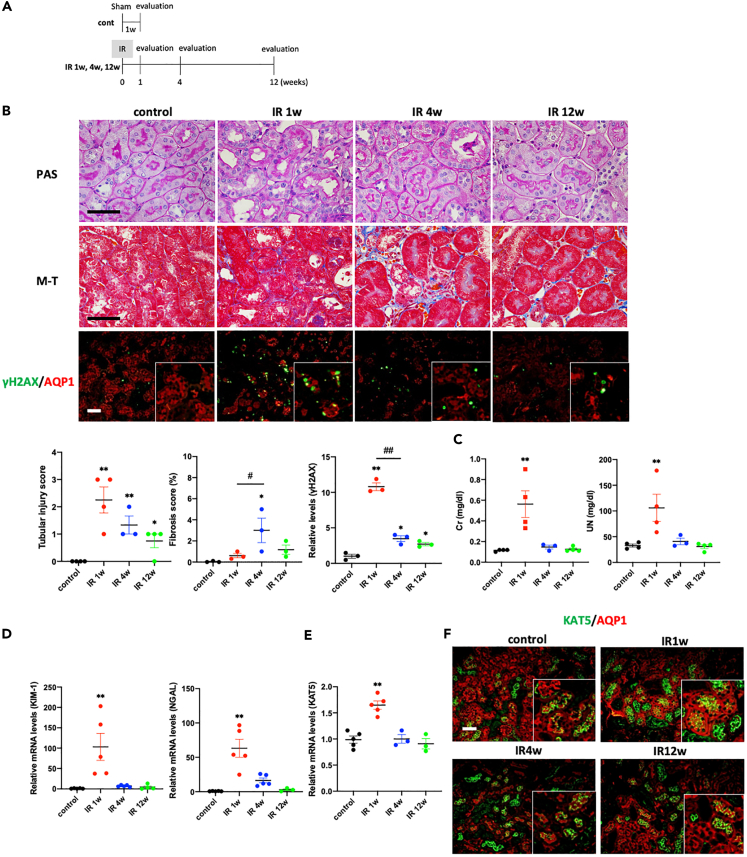
Time course of KAT5 expression and DNA damage repair following IR (A) Experimental protocol. Time course changes 1, 4, and 12 weeks after IR were evaluated (n = 3–5 per groups). (B) (Upper panels) Representative photomicrographs of PAS staining, Masson trichrome (M-T) staining, and immunofluorescence double staining of γH2AX (green)/AQP1 (red) in the kidney cortex. Scale bar, 50 μm. (Lower panels) Tubular injury scores in PAS staining, fibrotic area in M-T staining, and quantification of the immunolabeled double-positive area. (C) Serum creatinine and UN levels. (D) Expression of KIM-1 and NGAL in the kidney cortex. (E) Real-time RT-PCR analysis of KAT5 expression in the kidney cortex. (F) Representative photomicrographs of immunofluorescence double staining of KAT5 (green)/AQP1 (red) in kidney cortex. Scale bar, 50 μm. Data represent the mean ± SEM. ∗p < 0.05, ∗∗p < 0.01 versus controls, #p < 0.05, ##p < 0.01 versus the respective groups.

Next, the time course changes after IR were evaluated (Figure 2A). Following IR, the γH2AX-positive area was increased and then gradually decreased. The fibrotic area was slightly increased at 4 and 12 weeks after IR injury but it was less than 5% in this model (Figure 2B). At the time point, serum Cr and UN were recovered (Figure 2C), and KIM-1 and NGAL in the kidney cortex also returned to baseline levels (Figure 2D). Increased KAT5 expression at 1 week after IR was reduced to baseline at 4 and 12 weeks after IR (Figures 2E and 2F).

These results indicate that a sustained increase in KAT5 expression after preconditioning together with additional induction of KAT5 expression in secondary IR injury may contribute, at least in part, to the preconditioning effect.

### Knockdown of proximal tubular cell KAT5 attenuates the preconditioning effect

**Figure 3:**
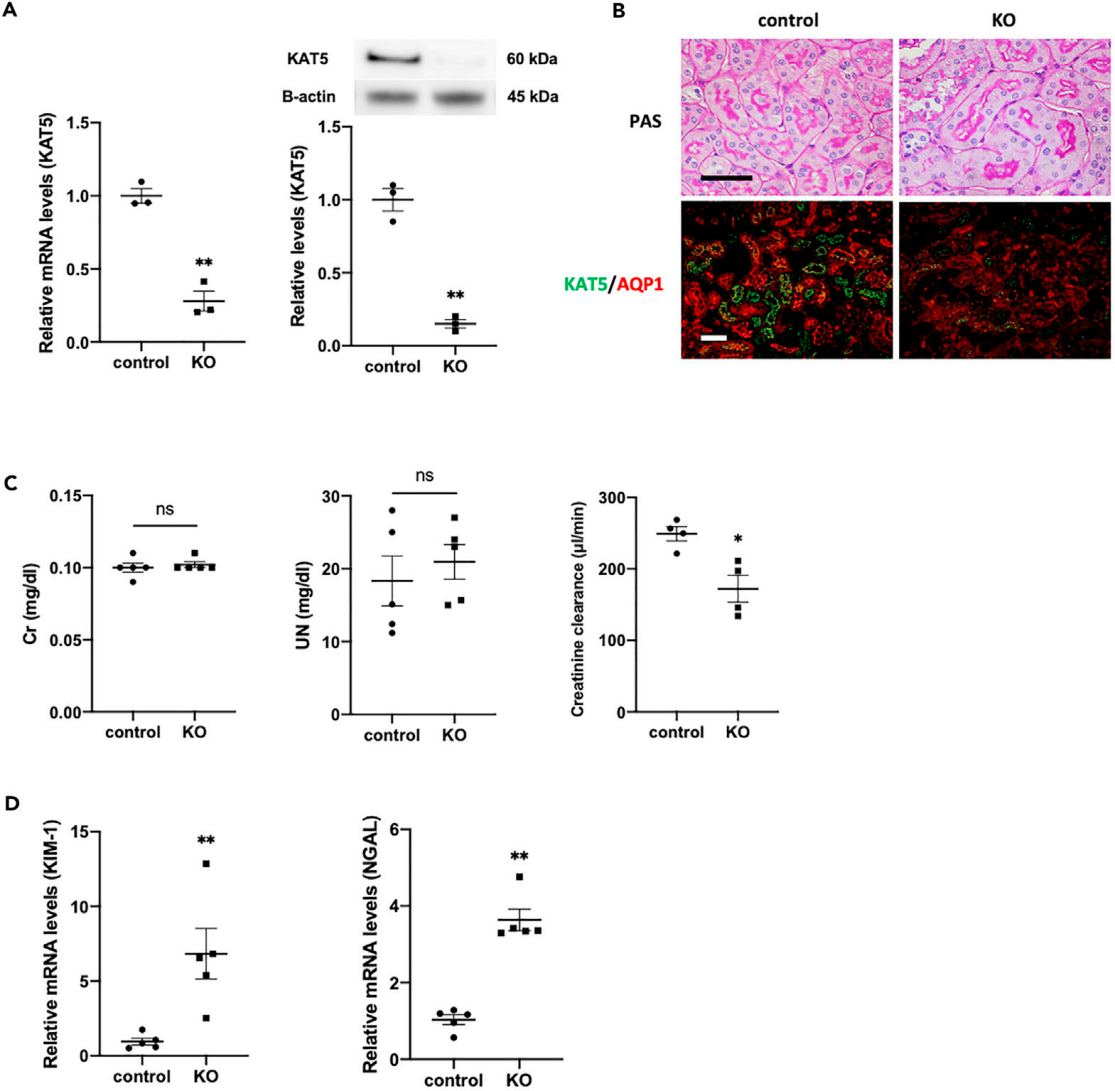
Phenotypes of proximal tubular-cell-specific KAT5 knockout mice (A) KAT5 expression in isolated proximal tubular cells of KOs and controls: (left) real-time RT-PCR; (right) western blots. (B) Representative photomicrographs of PAS staining and immunofluorescence double staining of KAT5 (green)/AQP1 (red) in the kidney cortex. Scale bar, 50 μm. (C) Serum creatinine, UN levels, and creatinine clearance. (D) Real-time RT-PCR analysis of KIM-1 and NGAL expression in the kidney cortex. Data represent the mean ± SEM. ∗p < 0.05, ∗∗p < 0.01 versus controls.

**Figure 4:**
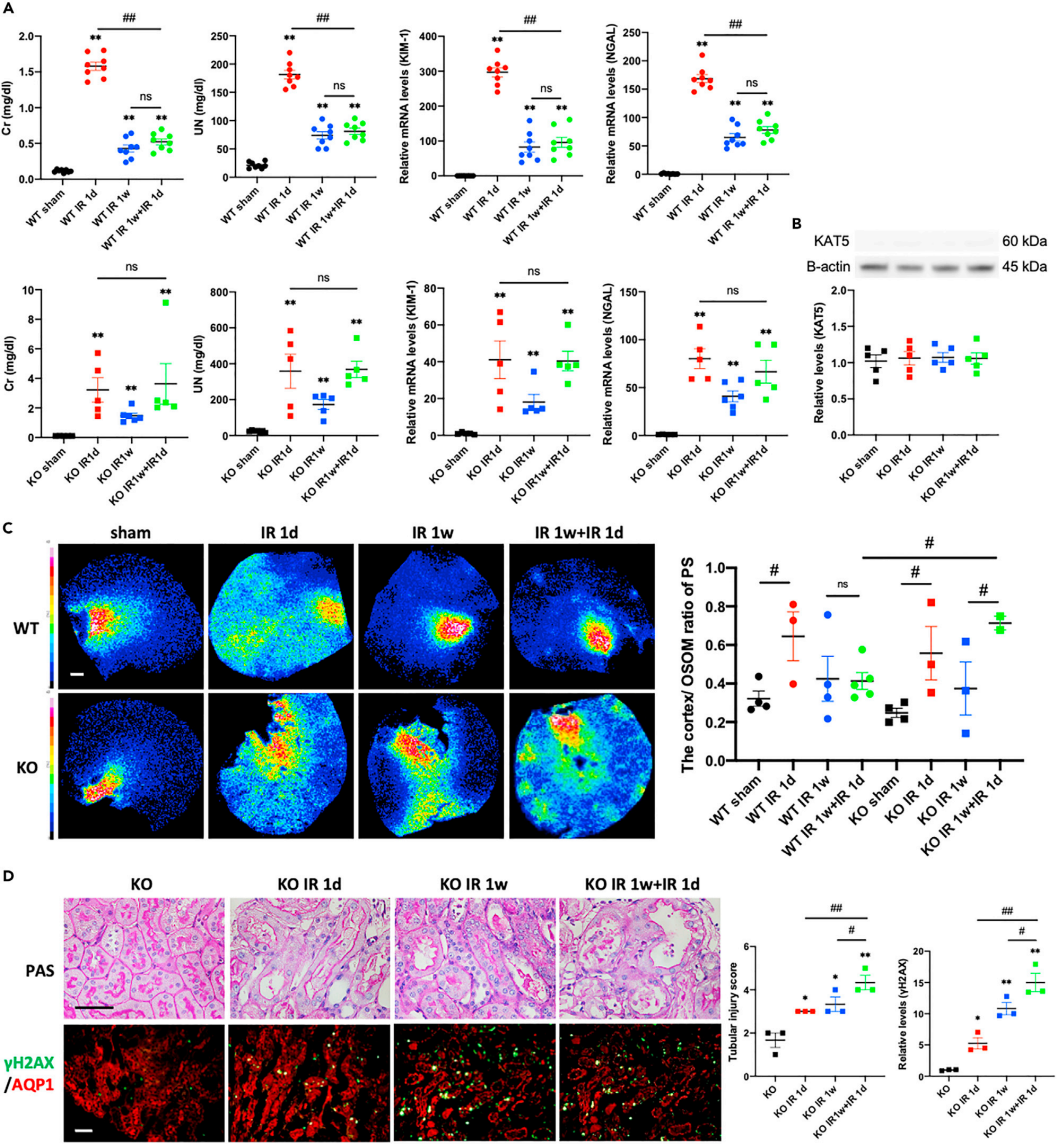
Knockdown of proximal tubular cell KAT5 attenuates the preconditioning effect IR injury with or without ischemic preconditioning was performed in the proximal tubular-cell-specific KAT5 KO mice (Kat5 fl/fl Cre+) and control littermates (Kat5 fl/fl Cre−) according to the experimental protocol as shown in Figure 1 (n = 3–5 per time point). (A) Serum creatinine, UN levels, and real-time RT-PCR analysis of KIM-1 and NGAL expression in the kidney cortex of controls and KOs following IR. (B) Western blot analysis of KAT5 expression in the isolated proximal tubular cells of KOs following IR. (C) Mass spectrometric images of phenyl sulfate (PS) (*m/z* 172.991) in snap-frozen murine kidney of KOs and controls. (Left panels) Representative heatmaps of MALDI-IMS. (Right panels) The cortex/outer stripes of outer medulla (OSOM) ratio of metabolites in the indicated groups. Scale bar, 500 μm. (D) (Left panels) Representative photomicrographs of PAS staining and immunofluorescence double staining of γH2AX (green)/AQP1 (red) in the kidney cortex. Scale bar, 50 μm. (Right panels) Tubular injury scores in PAS staining and quantification of the immunolabeled double-positive area. Data represent the mean ± SEM. ∗p < 0.05, ∗∗p < 0.01 versus controls, #p < 0.05, ##p < 0.01 versus the respective groups.

To evaluate the role of KAT5 expression in PT cells on the ischemic preconditioning effect, PT-specific KAT5 knockout (KO) mice (γGT-Cre + KAT5 ^flox/flox^) were generated. γGT is expressed in S1 to S3 of PT cells and is intensely expressed in S3 (Helbert et al., 1997). KAT5 expression was decreased to 10%–20% of wild-type control mice in isolated PT cells (Figure 3A), and this was confirmed by immunofluorescent staining (Figure 3B). Although there were no significant changes in the pathology, serum Cr, and UN (Figure 3B), KO mice showed decreased creatinine clearance (Ccr) (Figure 3C) and mild tubular damage with elevated KIM-1 and NGAL levels in the kidney cortex (Figure 3D). The levels of Cr and UN decreased one week after IR but remained higher than those of wild-type controls, suggesting prolonged kidney damage (Figure 4A). The preconditioning effect, evaluated by serum Cr and UN, and the expression of NGAL and KIM-1 almost vanished in the KAT5 KO mice (Figure 4A). KAT5 expression in the KO mice was not significantly increased following IR with preconditioning compared with IR without preconditioning (Figure 4B). The distribution of phenyl sulfate analyzed using MALDI-IMS, which is a potential marker for damaged kidneys, also showed attenuation of the preconditioning effect in KAT5 KO mice (Figures 4C and S2). The γH2AX level and TUNEL-positive area were increased in KAT5 KO mice compared with controls, and additional induction of PT cell damage was observed in IR even with preconditioning (Figures 4D and S3). These results indicate that KAT5 may play an important role in the preconditioning effect to protect PT cells from DNA damage.

### KAT5 is involved in the regulation of tubuloglomerular feedback following AKI with ischemic preconditioning

**Figure 5:**
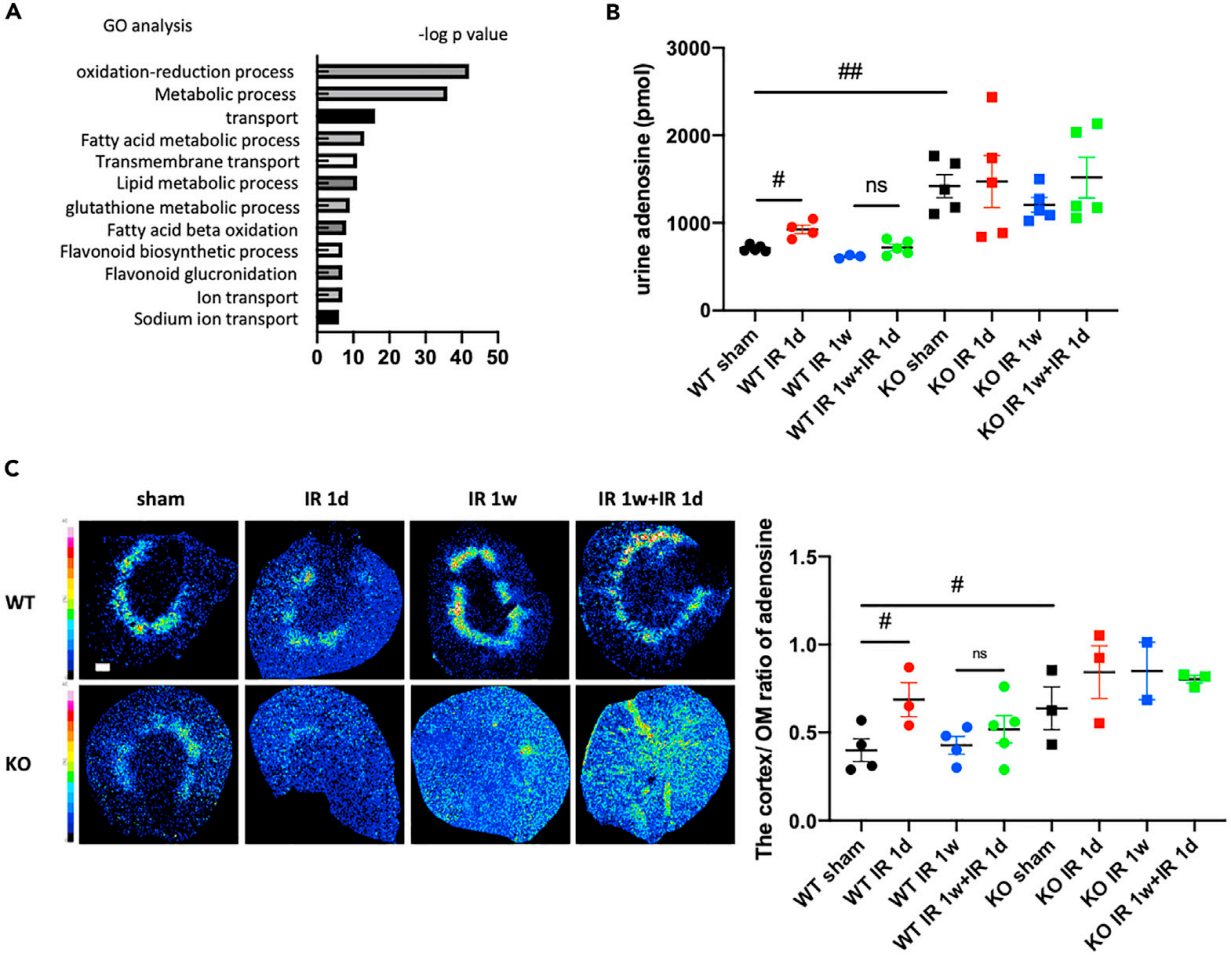
KAT5 is involved in the regulation of tubuloglomerular feedback (TGF) following AKI with ischemic preconditioning (A) Gene ontology analysis using isolated proximal tubular cells of KAT5 KO mice and controls. The graph shows -log p values calculated using Benjamini-Hochberg-corrected two-tailed t test for the enrichment of a specific pathway. (B) Urinary adenosine levels of the indicated groups (n = 3–5 per groups). (C) Mass spectrometric images of snap-frozen murine kidney of KOs and controls. (Left panels) Representative heatmaps of MALDI-IMS of adenosine (*m/z* 268.104) distribution. (Right panels) The cortex/outer medulla (OM) ratio of metabolites in the indicated groups. Scale bar, 500 μm. Data represent the mean ± SEM. #p < 0.05, ##p < 0.01 versus the respective groups.

It has been reported that TGF-induced afferent arteriole vasoconstriction, which is regulated by the chloride concentration of the macula densa, is a common hemodynamic insult in AKI models (Rein and Coca, 2019; Singh and Okusa, 2011). We next investigated the association of TGF regulation with the preconditioning effect because the KAT5 KO mice showed decreased Ccr at baseline, which may suggest PT cell KAT5-mediated regulation of glomerular filtration. In addition, microarray analysis using isolated PT cells in wild-type mice and KAT5 KO mice indicated that KAT5 in PT cells was involved in the regulation of ion channel expression as well as oxidation-reduction processes and lipid metabolism (Figure 5A).

As adenosine is the final-acting substance in TGF that mediates vasoconstriction of afferent arterioles, previous reports estimated TGF regulation using urinary adenosine levels (Kidokoro et al., 2019; Rajasekeran et al., 2017). It has been shown that urinary adenosine increase suggests the afferent arteriolar constriction and reduced hyperfiltration related to increased adenosine production by the macula densa. Urinary adenosine was significantly increased after IR without preconditioning, whereas they were decreased following IR with preconditioning. In KAT5 KO mice, urinary adenosine was significantly elevated compared with controls, which showed a sustained increase regardless of IR (Figure 5B). Furthermore, adenosine distribution in the kidney cortex was evaluated using MALDI-IMS. It was demonstrated that the ratio of the adenosine concentration in the kidney cortex/outer medulla was increased following IR without preconditioning, whereas it was not significantly changed after IR with preconditioning. On the other hand, adenosine distributions in KAT5 KO mice did not change, which showed a sustained increase compared with controls (Figures 5C and S4). These results suggest the involvement of TGF regulation in the preconditioning effect and that TGF is accelerated in KAT5 KO mice.

### KAT5-mediated increase in KCC3 expression is associated with the preconditioning effect via attenuation of TGF

**Figure 6:**
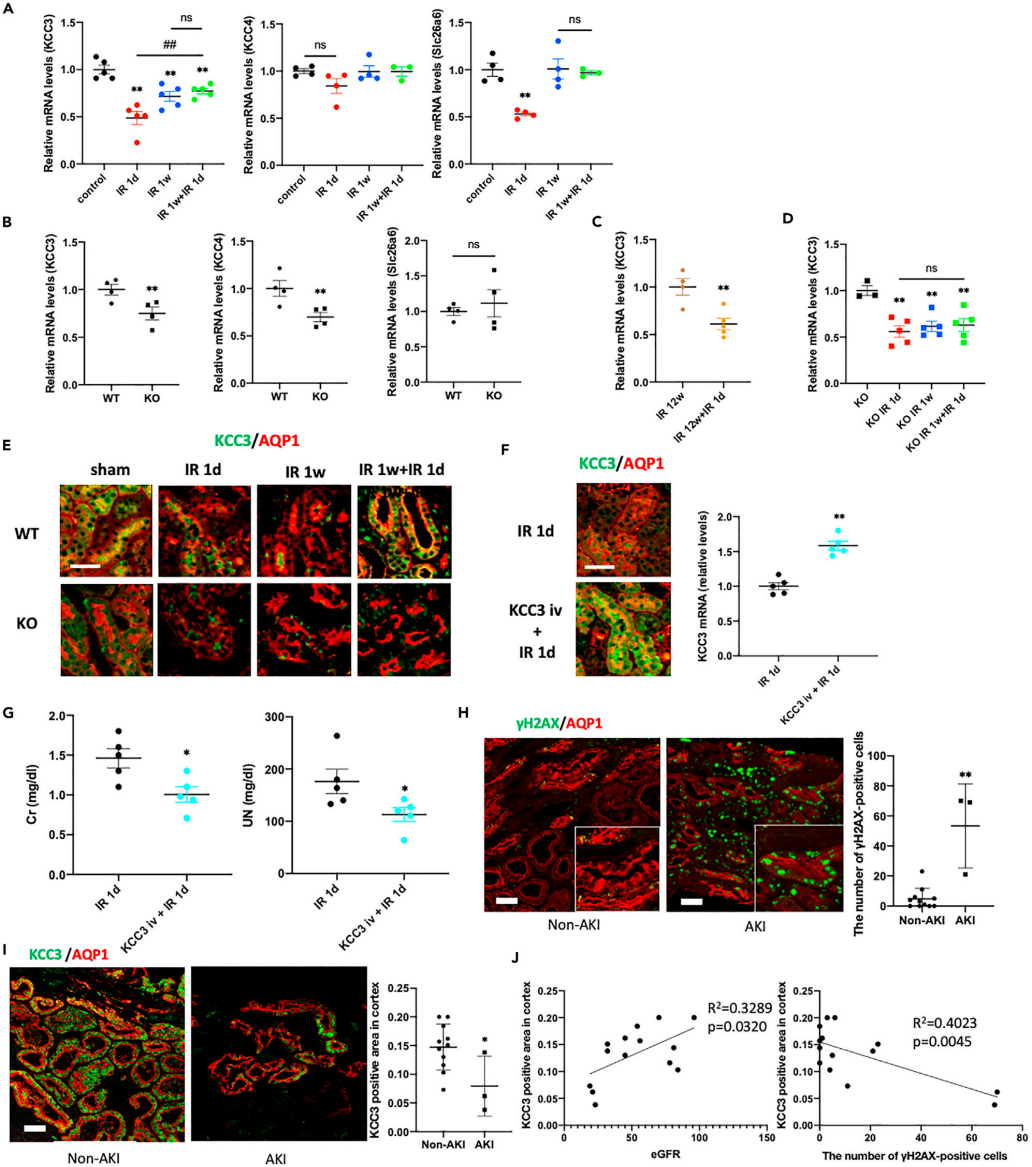
KAT5-mediated increase in KCC3 expression is associated with the preconditioning effect via attenuation of TGF (A) Real-time RT-PCR analysis of KCC3 (Slc12a6), KCC4 (Slc12a7), and Slc26a6 expression in the kidney cortex of wild-type control mice following IR according to the experimental protocol as shown in Figure 1 (n = 3–5 per groups). (B) Real-time RT-PCR analysis of KCC3 (Slc12a6), KCC4 (Slc12a7), and Slc26a6 expression in the kidney cortex of KOs and controls. (C) Real-time RT-PCR analysis of KCC3 expression in the kidney cortex following IR injury with preconditioning 12 weeks prior to the IR. (D) Real-time RT-PCR analysis of KCC3 expression in the kidney cortex following IR injury in KOs. (E) Representative photomicrographs of immunofluorescence double staining of KCC3 (green)/AQP1 (red) in kidney cortex. Scale bar, 50 μm. (F) (Left) Representative photomicrographs of immunofluorescence (IF) double staining of KCC3 (green)/AQP1 (red) 1 day after IR with or without KCC3 plasmid injection. (Right) Real-time RT-PCR analysis of KCC3 in the kidney cortex of 1 day after IR with or without KCC3 plasmid injection. (G) Serum creatinine and UN of WT mice 1 day after IR with or without KCC3 plasmid injection. (H) (Left panels) Representative photographs of immunofluorescent double staining of γH2AX (green) and AQP1 (red) in human biopsy samples with non-AKI or AKI. AKI, acute kidney injury. (Right panel) The number of γH2AX-positive cells in AQP1-positive PT cells. (I) (Left panels) Representative photographs of immunofluorescent staining of KCC3 (green) and AQP1 (red) in human biopsy samples with non-AKI or AKI. (Right panel) The ratio of KCC3 positive area/cortex except glomerular area. (J) (Left panel) Correlation between eGFR and the ratio of KCC3 positive area/cortex except glomerular area. (Right panel) Correlation between the number of γH2AX and AQP1 double-positive cells and the ratio of KCC3 positive area/cortex except glomerular area. The clinical data presented in Table S1. Scale bar, 50 μm. Data represent the mean ± SEM. ∗p < 0.05, ∗∗p < 0.01 versus controls, ##p < 0.01 versus the respective groups.

Next, we evaluated the role of chloride channel expression in PT cells, which is a determinant of chloride concentration in the macula densa. PT cells have several chloride channels, including slc12a6 (K-Cl cotransporter 3, KCC3) and slc12a7 (K-Cl cotransporter 4, KCC4) on the basal side and slc26a6 on the apical side of PT cells, regulating the chloride concentration of the macula densa. KCC3 and slc26a6 expression was significantly decreased in the kidney cortex 24 h after IR (Figure 6A). These results were confirmed using a recently published database of single-cell analysis (Rudman-Melnick et al., 2020) (Figure S5). Investigating the expression of the three chloride channels in KAT5 KO mice, we found that KCC3 and KCC4 expression was decreased (Figure 6B). Therefore, KCC3 action was our focus to clarify the mechanism of the KAT5-associated preconditioning effect through TGF regulation. Preconditioning for 1 week prior to IR attenuated the decrease in KCC3 expression. However, it was not affected by the preconditioning 12 weeks prior to IR injury (Figure 6C). Following IR with preconditioning, KCC3 expression was increased compared with IR without preconditioning in wild-type controls (Figures 6A and 6E). In KAT5 KO mice, cortex KCC3 expression was significantly reduced at baseline (Figure 6B), and preconditioning 1 week prior to IR did not induce KCC3 expression (Figures 6D and 6E).

We next examined the effects of restoring KCC3 expression in IR injury. We performed an *in vivo* gene transfer of KCC3-containing plasmids using a hydrodynamic-based gene transfer method. The efficacy of this gene transfer method has been previously reported (Bu et al., 2011; Hayashi et al., 2014; Hishikawa et al., 2019). We confirmed the increase in tubular KCC3 expression 1 day after injection (Figure 6F). Transient KCC3 upregulation alleviated elevation of serum Cr and UN (Figure 6G), suggesting the importance of KCC3 in AKI prevention.

Together with the results of accelerated TGF in KAT5 KO mice, these results indicate that KCC3 may be involved in the KAT5-mediated preconditioning effect of AKI through TGF regulation.

Next, changes in KCC3 expression caused by tubular damage in humans were examined using human biopsy samples. The profile of the patients is shown in Table S1. The number of γH2AX-positive cells was significantly increased in the AQP1-positive PT cells of patients with AKI compared with those of non-AKI patients (Figure 6H). KCC3 expression, which was reported to be expressed specifically in PT cells in the kidney, was decreased in the kidney cortex of patients with AKI compared with those with non-AKI (Figure 6I). The number of γH2AX and AQP1 double-positive cells was negatively associated with eGFR (r^2^ = 0.4151, p = 0.0129). KCC3 expression was significantly correlated with eGFR as well as the number of γH2AX and AQP1 double-positive cells (Figure 6J). These results indicate that a decrease in KCC3 expression is associated with PT cell damage and decreased eGFR in humans.

### KAT5 regulates KCC3 expression through an epigenetic mechanism

**Figure 7:**
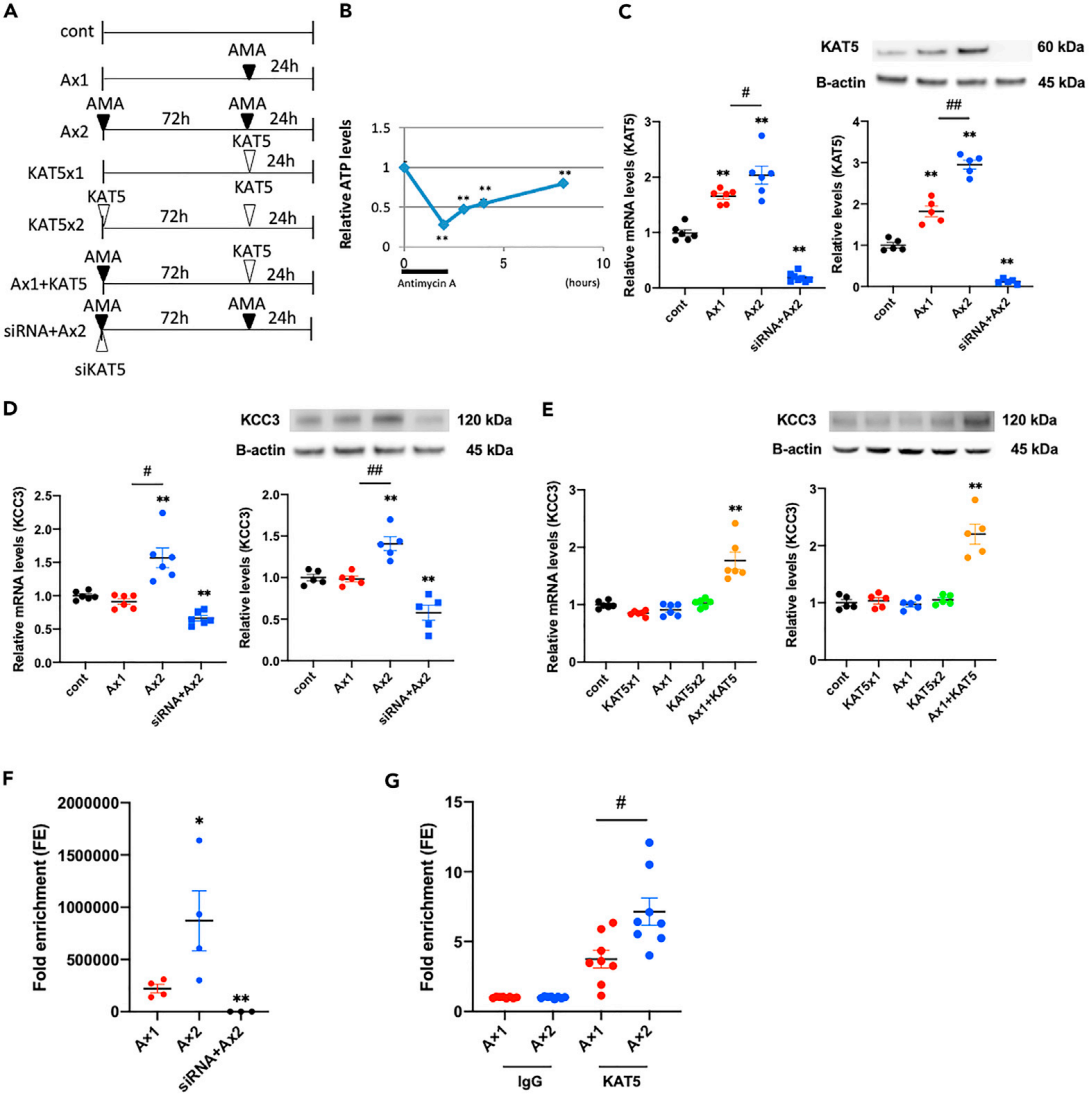
KAT5 regulates KCC3 expression through an epigenetic mechanism *In vitro* analysis using cultured human tubular epithelial cells (HK2 cells). (A) Experimental protocol. HK2 cells were treated with antimycin A (AMA) for 1 h and analyzed 24 h after AMA treatment without (Ax1) or with (Ax2) the pretreatment of AMA 72 h prior to the injury. pCMV-KAT5 expression plasmid was transfected to AMA-treated HK2 cells 72 h after AMA treatment and analyzed 24 h after transfection (Ax1+KAT5). KAT5 knockdown experiment was performed using siRNA before the AMA treatment (siRNA + Ax2). (B) Time course of ATP levels after AMA treatment. Decreased ATP levels after AMA treatment gradually recovered and reached plateau almost 8 h after starting the treatment. (C) KAT5 expression of indicated groups: (left) real-time RT-PCR; (right) western blots. (D and E) KCC3 expression of indicated groups: (left) real-time RT-PCR; (right) western blots. (F) Chromatin accessibility assay of the KCC3 promoter region. (G) Quantitative ChIP assay performed using IgG and KAT5 antibodies. Data was normalized to input fraction, and the results were relative to that of IgG which was set 1. Data represent the mean ± SEM. ∗p < 0.05, ∗∗p < 0.01 versus controls. #p < 0.05 versus the respective groups, ##p < 0.01 versus the respective groups.

Then, we investigated the mechanism by which KAT5 regulates KCC3 expression in proximal tubular damage *in vitro* using HK2 cells. First, HK2 cells were subjected to ATP depletion by antimycin A (AMA) (Figures 7A and 7B), as reported previously (Yoshino et al., 2003). KAT5 expression was more enhanced at the second ATP depletion than at the first ATP depletion (Figure 7C), similar to the *in vivo* ischemic AKI model. KCC3 expression was elevated following the second treatment with AMA (Figure 7D). Knockdown of KAT5 using siRNA blocked an increase in KCC3 expression following the second treatment with AMA (Figures 7C and 7D), which suggests the involvement of KAT5 in regulating KCC3 expression. Interestingly, simple KAT5 transfection did not cause altered KCC3 expression and showed only a small increase even after the second transfection of KAT5; however, KAT5 transfection following AMA treatment significantly induced increased KCC3 expression (Figure 7E). These results indicated that KAT5 plays an important role in regulating KCC3 expression, although other KCC3 expression regulation mechanisms caused by AMA treatment needs to be uncovered.

Based on the results, we focused on the chromatin accessibility of the KCC3 promoter region (−352 to −144), as shown in Figure S6A. The chromatin accessibility was increased following the second AMA treatment compared with the first treatment (Figure 7F). Moreover, KAT5 knockdown caused a decrease in chromatin accessibility even after the second AMA treatment, suggesting the involvement of KAT5 in maintaining chromatin accessibility. In addition, ChIP analysis revealed that KAT5 binding to the KCC3 promoter region was significantly increased after the second injury of PT cells (Figure 7G). These results indicate that elevated KCC3 expression following the second injury was caused by increased binding of KAT5 to the KCC3 promoter region, which was enabled by the enhanced accessibility of chromatin. ChIP analysis using γH2AX antibody showed that DNA DSB sites in the KCC3 promoter region were not significantly increased at the second AMA injury compared with the first injury (Figure S6B). No significant changes in the DNA methylation of the KCC3 promoter region were observed between the first and second AMA treatments (Figure S6C). These results indicated that KAT5 regulated KCC3 expression following AKI through attenuation of additional DNA damage induction, maintaining chromatin accessibility and binding to the KCC3 promoter region.

## Discussion

**Figure 8:**
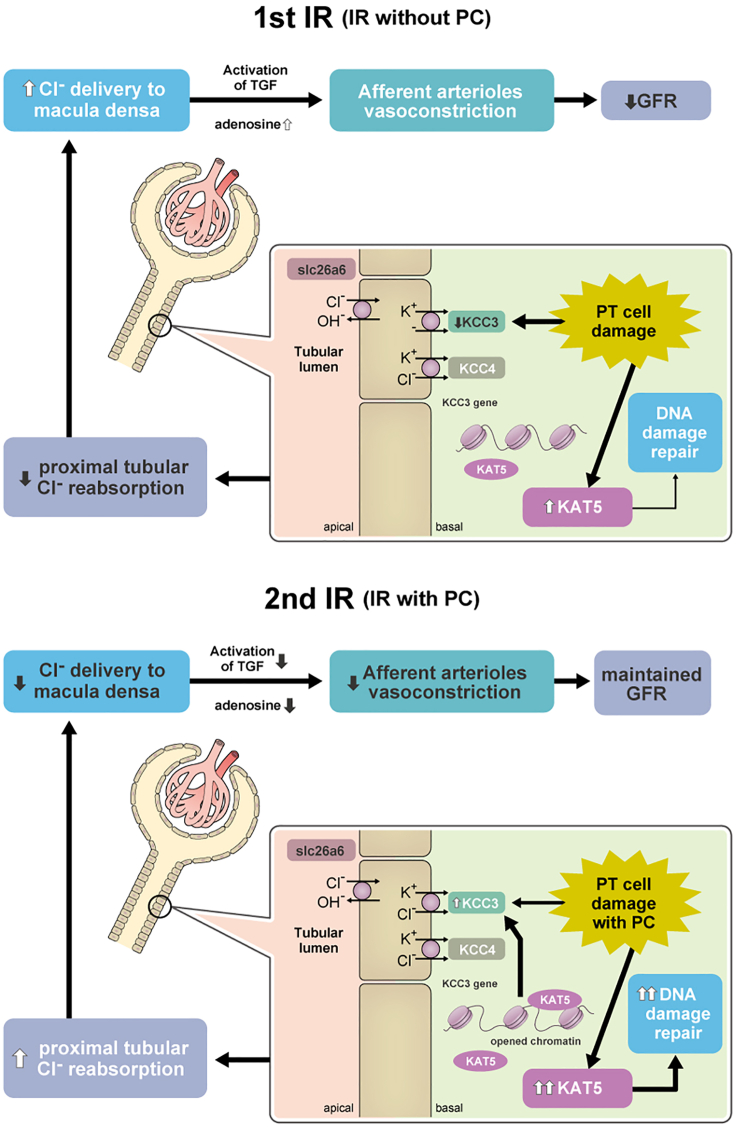
A scheme of the ischemic preconditioning effect via KAT5 (Upper panel) IR injury causes proximal tubular (PT) cell damage, which induces a decrease in KCC3 expression as well as KAT5-mediated DNA damage repair, leading reduced Cl^−^ reabsorption in PT cells and increased Cl^−^ delivery to macula densa. It triggers activation of TGF via elevated adenosine concentration, which causes vasoconstriction of afferent arterioles and decreased GFR. (Lower panel) When IR injury occurs following preconditioning, promoted KAT5 expression causes accelerated DNA damage repair and protection from apoptosis as well as restoration of KCC3 expression through epigenetic mechanism, which induces increased Cl^−^ reabsorption in PT cells and reduced Cl^−^ delivery to macula densa. It causes attenuation of TGF activation with reduced adenosine concentration and maintained GFR. IR: ischemic reperfusion, PC: preconditioning, GFR: glomerular filtration rate.

In the present study, we demonstrated that the DNA repair factor KAT5 mediated the preconditioning effect of AKI not only through cell protection but also through attenuation of the TGF response via epigenetic regulation of chloride channel KCC3 expression in PT cells, as summarized in Figure 8.

Preconditioning effect was first described in the kidney by Zager and Baltes (Zager and Baltes, 1984), and the effect persists from several days to several weeks in mouse models (Jang et al., 2012; Kolb et al., 2018; Wever et al., 2012). We confirmed that the preconditioning one week prior to IR injury was effective, using an IR model of bilateral renal arteries in mice. Traditionally, the preconditioning effect is thought to be mediated by various factors, including glucose and mitochondrial metabolism, inhibition of reactive oxygen species (ROS) production and inflammation, and suppression of proapoptotic molecules (Basile et al., 2012; Kapitsinou and Haase, 2015). This study demonstrated that enhanced DNA repair due to elevated expression of DNA repair factors, including KAT5, was associated with a preconditioning effect. Given a recent report that following PT cell injury, DNA repair rather than cell proliferation plays the central role in recovery and longevity (Kishi et al., 2019), our results may be reasonable, which suggested that more rapid and efficient DNA repair attenuated additional kidney damage in the second IR.

In this study, MALDI-IMS was adopted to examine the distribution of the molecules in the kidney, which is a useful method in that the dynamics in the kidney can be visualized (Fujii et al., 2019; Yamamoto et al., 2014). We analyzed the distribution of the uremic toxin phenyl sulfate (PS), known as a serum marker associated with kidney damage (Kikuchi et al., 2019), which accumulates in the kidney cortex following IR injury. The accumulation of PS in the cortex as well as increased DNA damage and apoptosis in PT cells of KAT5 KO mice with preconditioned IR suggested that KAT5 contributed to the preconditioning effect, at least in part, through protection of PT cells.

Recently, it has been reported that deficiencies in DNA repair factors result in nephronophthisis (Airik et al., 2014; Chaki et al., 2012; Choi et al., 2013; Jain et al., 2019; Zhou et al., 2012). Genome-wide transcriptomic analysis in PT cells revealed several deregulated pathways that could contribute to the nephronophthisis phenotype, including alterations in anion transport (Jain et al., 2019). Therefore, we next investigated whether KAT5 might be involved in electrolyte regulation. The microarray analysis results and decreased CCr in the KAT5 KO mice supported the role of the DNA repair factor KAT5 in PT cells in the regulation of glomerular filtration through the expression of ion channels, although the result of microarray might reflect the indirect involvement of KAT5 in PT cells for regulation of ion channel expression.

TGF is a mechanism by which the kidney regulates the glomerular filtration rate (GFR) in which an increased distal tubular sodium chloride concentration causes a basolateral release of adenosine from macula densa cells, leading to vasoconstriction of afferent arterioles. Injury to the proximal tubule in AKI impairs solute reabsorption and increases distal solute delivery. TGF-mediated afferent arteriole vasoconstriction is commonly observed in various AKI models (Singh and Okusa, 2011). Although TGF-mediated decreases in GFR and oliguria primarily play a protective role by minimizing volume loss during AKI (Rein and Coca, 2019), prolonged afferent arteriole vasoconstriction can lead to ischemia and additional tubular injury (Oken, 1984), which inhibits the reabsorption of solutes. To our knowledge, this is the first report describing a possible association of an attenuated TGF with ischemic preconditioning by altered distribution of adenosine in murine kidney and urinary adenosine levels.

KCC3 is an integral membrane protein that regulates intracellular chloride concentrations. Kidney KCC3 expression is specific to the proximal tubule at the basolateral membrane of S1 to S3 (Boettger et al., 2003; Jentsch, 2005; Mercado et al., 2005). Mutation of KCC3 causes a severe peripheral neuropathy called Andermann syndrome in humans, and KCC3 knockout mice show not only severe neurodegeneration but also arterial hypertension, the mechanism of which has not been elucidated (Boettger et al., 2003; Howard et al., 2002). Although it has been reported that proximal tubular KCC3 expression is induced by hyperglycemia (Melo et al., 2013), how KCC3 expression is altered after tubular damage in AKI has not been clarified. This study revealed that KCC3 expression in the kidney cortex was decreased 24 h after IR in a mouse model and was confirmed to be decreased in tubular DNA damage in human biopsy samples. In septic AKI, proinflammatory cytokines cause a downregulation of renal chloride channels (Schmidt et al., 2007), which could generate increased distal delivery of chloride and initiate the TGF reaction. Decreased KCC3 expression in the cortex may be caused by downregulation of KCC3 expression in PT cells as well as loss of PT cells.

*In vitro* analysis suggests that KAT5 regulates KCC3 expression through an epigenetic mechanism. KAT5 may be involved in attenuating additional DNA damage induction, maintaining chromatin accessibility, and binding to the KCC3 promoter region. A sustained increase in KAT5, a histone acetyltransferase, may induce chromatin opening through histone acetylation, although factors other than KAT5 may be necessary for chromatin opening in the KCC3 promoter region. However, additional factors that may act together with KAT5 to maintain the chromatin accessibility could not be uncovered in this study. In addition, altered DNA methylation was not observed in the KCC3 promoter region between the first and second injuries. In our previous study in glomerular podocytes with diabetic nephropathy, KAT5-mediated DNA repair was associated with decreased DNA methylation. This may be due to the difference between chronic injury, such as hyperglycemia, and transient damage caused by temporary AMA treatment. Since DNA methylation is a sustained epigenetic marker, it may be a reason why the preconditioning effect is a relatively short period of AKI memory. In this study, we focused on the expression of chloride channels; however, there is a possibility that KAT5 may also regulate the expression of other ion channels, such as sodium transporters, which are involved in TGF regulation during AKI through sodium delivery to the distal tubules (Rein and Coca, 2019).

In conclusion, this study demonstrated a novel mechanism of the preconditioning effect mediated by the promotion of DNA repair and attenuation of TGF through KAT5. Furthermore, maintaining chromatin accessibility of the KCC3 promoter region with increased KAT5 expression contributes to the preconditioning effect, which is one of the suggested mechanisms of AKI memory. This study suggests an important aspect of a DNA repair factor that it plays a multitasking role, not only in the DNA repair of damaged cells but also in regulation of remote hemodynamic changes, which could affect the organ function. It may support the fact that DNA repair is cell-type specific and suggest the possibility of DNA repair factors as organ- and cell-specific therapeutic targets. A better understanding of DNA repair factors in various cell types is necessary to investigate a novel strategy for disease therapy.

### Limitations of the study

Our study demonstrated that proximal tubular cell KAT5 may act against IR injury through promoted DNA repair and regulation of TGF via KCC3 expression. Although we investigated the regulation of TGF by MALDI-IMS and urinary adenosine, it is better to evaluate the glomerular hemodynamic function using *in vivo* imaging. A future study is necessary for precise understanding of the TGF regulation.

## STAR★Methods

### Key resources table

**Table:** 

REAGENT or RESOURCE	SOURCE	IDENTIFIER
**Antibodies**		

Anti-KAT5 antibody (IF, ChIP)	Santa Cruz Biotechnology	Cat# sc-25378, RRID:AB_2280606
Anti-KAT5 antibody (WB)	Proteintech	Cat# 10827-1-AP, RRID:AB_2128431
Anti-γH2AX antibody (IF)	Cell Signaling Technology	Cat# 9718, RRID:AB_2118009
Anti-γH2AX antibody (ChIP)	NOVUS	Cat# NB100-384, RRID:AB_10002815
Anti-KCC3 antibody (IF)	Bioss	Cat# BS-11952R
Anti-KCC3 antibody (WB)	NOVUS	NBP1-06044, RRID:AB_1555481
Anti-AQP1 antibody	Abcam	ab9566, RRID:AB_296494
Anti-beta-actin antibody	Cell Signaling Technology	Cat# 4967, RRID:AB_330288

**Biological samples**		

Human kidney samples	Keio University School of Medicine	N/A

**Chemicals, peptides, and recombinant proteins**		

Lipofectamine™ 2000 Transfection Reagent	Thermo Fisher Scientific	Cat# 11668019
Lipofectamine™ RNAiMAX Transfection Reagent	Thermo Fisher Scientific	Cat# 13778150
RIPA Lysis Buffer System	Santa Cruz Biotechnology	Cat# sc-24948
ISOGEN	NIPPON GENE	Cat# 319-90211
TaqMan Reverse Transcription Reagents	Invitrogen	Cat# N8080234

**Critical commercial assays**		

NucleoSpin Tissue kit	MACHEREY-NAGEL	Cat# 740952.50
EpiTect Bisulfite Kit	Qiagen	Cat# 59104
EZ-ChIP	Millipore	Cat# 17-295
EpiQuik Chromatin Accessibility Assay Kit	Epigentek	Cat# P-1047-48

**Deposited data**		

Mouse IRI scRNA	CINCINNATI CHILDREN'S HOSPITAL MEDICAL CENTER	https://research.cchmc.org/PotterLab/scIRI/
Microarray data	Gene Expression Omnibus database	https://www.ncbi.nlm.nih.gov/geo/query/acc.cgi Accesion number GSE168535

**Experimental models: Cell lines**		

HK-2 cells	ATCC	Cat# CRL-2190, RRID:CVCL_0302

**Experimental models: Organisms/strains**		

C57BL/6J	Sankyo Laboratories	N/A

**Oligonucleotides**		

See Table S2	This paper	N/A

**Recombinant DNA**		

Slc12a6 (NM_133648) Mouse Tagged ORF Clone	ORIGENE	Cat# MR226241
pCMV-SPORT6-KAT5	RIKEN Center for Life Science Technologies	Cat# HGX056147
pCMV-SPORT6	RIKEN Center for Life Science Technologies	N/A

**Software and algorithms**		

Photoshop CC 2018 software	Adobe	N/A
Statview 5.0	SAS Institute	N/A

### Resource availability

#### Lead contact

Further information, requests, and inquiries should be directed to and will be fulfilled by the Lead Contact, Kaori Hayashi (kaorihayashi@keio.jp).

#### Materials availability

The study did not generate new unique reagents.

### Experimental model and subject details

#### Ischemic reperfusion models

Ischemic reperfusion was performed in 8-week-old male C57BL/6J mice obtained from Sankyo Laboratories (Tokyo, Japan) by clamping the bilateral renal arteries for 30 minutes followed by reperfusion. Briefly, mice were anesthetized with ketamine/ xylazine (100/10 mg/kg) i.p. injections and placed on a heating pad to maintain body temperature. Both renal pedicles were clamped for 30 minutes followed by reperfusion, which was confirmed visually upon release of the clamps. As a control, sham-operated animals were subjected to the same surgical procedure except for clamping of the renal pedicles. After the wounds were closed, the mice were kept on the heating pad until they regained consciousness.

#### Generation of PT cell-specific KAT5 knockout mice

The generation of KAT5^flox/flox^ mice has been described previously (Hishikawa et al., 2019). The accession no. of the KAT5 (loxP) mouse is CDB1329K (http://www2.clst.riken.jp/arg/mutant%20mice%20list.html). γGT-cre mice were generously provided by Eric Nelson at Vanderbilt University School of Medicine (Jackson Laboratory). Proximal tubular cell-specific *KAT5* knockout (KO) mice were produced by crossing KAT5^flox/flox^ mice on a C57BL/6J background with γ-GT-Cre mice. γ-GT-Cre mice on a C57BL/6J background have demonstrated proximal tubular cell-specific Cre recombinase activity (Ranganathan et al., 2014).

#### Human kidney samples

Kidney samples were obtained from patients with minor glomerular abnormalities (MGAs), depending on kidney biopsy findings with basal techniques, including light microscopy. The AKI group included patients diagnosed clinically and pathologically, with tubular injury recognized by skilled pathologists. Characteristics of the subjects are shown in Table S1. Biopsy samples were with informed consent from the patients and in accordance with the Declaration of Helsinki.

#### Cell lines

HK-2 cells, human immortalized proximal tubular epithelial cells (ATCC, CRL-2190™) were maintained at 37°C with 5% CO2 in keratinocyte serum-free medium (Keratinocyte-SFM, Gibco Life Technologies), supplemented with epidermal growth factor (EGF; 5 ng/ml) and bovine pituitary extract (40 pg/ml).

#### Study approval

All animal studies were performed in accordance with the animal experimentation guidelines of Keio University School of Medicine and RIKEN, and the protocols were approved by the Laboratory Animal Center of Keio University School of Medicine and the Institutional Animal Care and Use Committee of RIKEN Kobe Branch. For the human studies, biopsy samples were obtained from renal biopsies at Keio University Hospital with informed consent from the patients and in accordance with the Declaration of Helsinki, approved by the Ethics Committee of Keio University School of Medicine (approval number: 20180159).

### Method details

#### Biochemical studies

Mice were housed in a pathogen-free environment under controlled conditions (temperature 20-26°Cm humidity 40-70%, light/dark cycle 12h/12h). For urine collection, mice were individually housed in metabolic cages for 24 hours. Urinary Cr levels and serum Cr, UN levels were measured enzymatically using an automated chemistry analyzer (LSI Medience, Tokyo, Japan). Urinary adenosine levels were measured using adenosine deaminase followed by a multistep enzymatic approach (Adenosine Assay Kit; Abcam).

#### Histological studies

The kidneys were removed, fixed in 4% paraformaldehyde and then embedded in paraffin blocks. Paraffin sections were stained with periodic acid-Schiff (PAS), Masson trichrome (MT) or a combination of KAT5, KCC3 or γH2AX and AQP1 antibodies, followed by incubation with a secondary antibody for immunofluorescent staining. Stained paraffin sections were photographed using a laser confocal microscope (LSM710; Zeiss), and five ×20 field images were acquired and quantified using color channel analysis and pixel counting using Photoshop CC 2018 software (Adobe) as previously described (Hayashi et al., 2014). The mean value of the control samples was assigned an arbitrary unit of 1. Tubular injury was scored as previously described (Coca et al., 2012; Kang et al., 2001), according to the following scoring system: 0: no tubular injury; 1: ≤10% of tubules injured; 2: 11%–25% of tubules injured; 3: 26%–50% of tubules injured; 4: 51%–74% of tubules injured; and 5: ≥75% of tubules injured.

#### PT cell isolation

PT cells were isolated by means of a previously described protocol with modifications (Legouis et al., 2015). Briefly, following endothelial cell depletion using the anti-CD31 antibody (BL102503; Biolegend), PT cells were isolated from minced murine kidneys using magnet-activated cell sorting with an anti-Prominin1 antibody (Miltenyi Biotec).

#### Gene transfer experiments

The KCC3 expression plasmid (MR226241; ORIGENE) or control vector (1 mg/kg) was administered systemically by rapid tail vein injection, as previously described, for hydrodynamics-based *in vivo* gene transfer (Bu et al., 2011; Hayashi et al., 2014; Hishikawa et al., 2019).

The plasmid was administered just before the ischemic reperfusion.

#### Cell treatment

HK-2 cells, human immortalized proximal tubular epithelial cells (ATCC, CRL-2190™) were used in *in vitro* studies. To induce proximal tubular cell damage, ATP depletion was performed using antimycin A (AMA, Sigma) by means of a previously described protocol (Yoshino et al., 2003). A confluent monolayer of HK-2 cells was incubated in PBS with 1.5 mM CaCl_2_, 2 mM MgCl_2_, and 1 μM AMA for 60 minutes. During the recovery phase after the injury, cells were repleted with ATP by incubation with regular growth medium. Cellular ATP levels were determined with a luciferase-based assay kit (Sigma). pCMV-SPORT6-KAT5 and pCMV-SPORT6 were purchased from the RIKEN Center for Life Science Technologies (Hyogo, Japan) and transfected into cultured HK2 cells using Lipofectamine (Invitrogen) according to the manufacturer’s instructions. For KAT5 knockdown experiments, commercially synthesized siRNA oligonucleotides (Invitrogen, listed in Table S2) and control siRNA (HiGC; 12935-400) were transfected into cultured HK2-cells using Lipofectamine RNAiMAX (Invitrogen) according to the manufacturer’s instructions.

#### Real-time quantitative PCR, PCR array and microarray

Following extraction of mRNA from the kidney cortex, isolated PT cells or HK-2 cells, gene expression was quantitatively analyzed with real-time RT-PCR by the delta–delta-cycle threshold (Ct) method (ΔΔCt) using the primers and probes described in Table S2. To determine DNA repair gene changes following IR with or without preconditioning, a PCR array (RT^2^ Profiler PCR Arrays, QIAGEN) was performed. RNA samples from isolated PT cells in wild-type mice or KAT5 KO mice were subjected to expression analysis on Clariom S mouse arrays (Affymetrix) according to Affymetrix standard protocols. The microarray gene expression data are available at the Gene Expression Omnibus database under the accession number GSE168535.

#### Western blot analysis

The kidney cortex was homogenized in RIPA lysis buffer with a protease inhibitor cocktail (Invitrogen). Proteins were resolved using sodium dodecyl sulfate-polyacrylamide gel electrophoresis (SDS-PAGE), transferred onto polyvinylidene difluoride (PVDF) membranes and detected using the antibodies listed in key resources table.

#### Bisulfite genomic sequencing (BGS)

DNA was extracted with the NucleoSpin Tissue kit (Takara, Japan). Bisulfite conversion of fragmented purified DNA was performed using the EpiTect Bisulfite Kit (Qiagen). Bisulfite-modified DNA was amplified by PCR with BGS primers as described in Table S2. Amplified PCR products were cloned into the TA cloning vector pT7-Blue and sequenced. The analyzed murine KCC3 promoter region is presented in Figure S6A, and 5 CpGs (CpGs 1–5) were included in the region.

#### ChIP assay

The ChIP assay was performed using a commercially available kit according to the manufacturer’s instructions (EZ-ChIP; Millipore). The antibodies used for analysis are described in key resources table.

#### Chromatin accessibility

KCC3 gene promoter accessibility was determined using the EpiQuik Chromatin Accessibility Assay Kit (P-1047-48, Epigentek) following the protocol provided by the manufacturer as described previously (Hishikawa et al., 2019). Chromatin was isolated from HK-2 cells with AMA treatment and treated with a nuclease (Nse) mix. DNA was then isolated and amplified using quantitative PCR and gene-specific primers for KCC3 (listed in Table S2 and Figure S6A). Control primers were provided to determine the successful digestion of the chromatin. The fold enrichment (FE) was calculated by the ratio of amplification efficiency of the Nse-treated DNA sample over that of a control sample that was not treated with a nuclease (no Nse). The fold change in enrichment was calculated using the formula FE = 2^(NseCT – no NseCT)^ ×100%. FE% >1600% indicates that the gene region is in the opened chromatin, while FE% <400% represents that the gene region is in closed chromatin.

#### MALDI-IMS

A MALDI-quadrupole ion trap (QIT)-time of flight (TOF) mass spectrometer (iMScope: Shimadzu Corp, Kyoto, Japan) was used for data acquisition. In the current study, frozen kidney tissues were dissected and cryosections with a 10-μm thickness prepared using a cryostat (CM 3050S; Leica Microsystems, Wetzlar, Germany). Sections were thaw-mounted on indium tin oxide (ITO)- and MAS-coated slides (#SI0100M, Matsunami Glass, Osaka, Japan) and dried in silica gel-containing plastic tubes. Slides were then sprayed with 5 mg/mL 9-aminoacridine (Merck Millipore, Germany) in 80% ethanol for negative ion detection or 30 mg/mL α-cyano-4-hydroxycinnamic acid (Sigma-Aldrich, USA) in 75% methanol for positive ion detection. Mass images were acquired in positive (m/z 100-450) and negative (m/z 85-400) ion modes, with 80 laser shots per spot. Mass spectra acquired from each measurement point in a 25-μm pitch were reconstructed as two-dimensional heat maps using IMAGEREVEAL MS software (Shimadzu Corp, Kyoto, Japan). After raster scanning for IMS, sections were washed with acetone and stained with hematoxylin and eosin (HE) according to the manufacturer’s protocol (Muto Pure Chemicals, Tokyo, Japan). HE-stained slides were scanned with a virtual slide scanner (Nanozoomer-XR, Hamamatsu Photonics, Hamamatsu, Japan). The renal cortex, the outer stripes of the outer medulla (OSOM), the inner stripes of the outer medulla (ISOM) and the inner medulla (IM) were identified in each image.

### Quantification and statistical analysis

The results are expressed as the mean±SEM. Statistical comparisons were made by ANOVA followed by Scheffe’s post hoc test between more than 2 groups and by a 2- tailed Student’s t-test between 2 groups. Statistical significance was defined as P<0.05.

All statistical details of experiments can be found in the figure legends. Statview 5.0 software (SAS Institute) was used.

## Data Availability

The microarray gene expression data are available at the Gene Expression Omnibus database under the accession number GSE168535.
