# Supplementary material for: DNA repair factor KAT5 prevents ischemic acute kidney injury through glomerular filtration regulation
Source: iScience. 2021 Nov 14;24(12):103436. doi: 10.1016/j.isci.2021.103436 (PMC8633972; doi:10.1016/j.isci.2021.103436)
Supplement: Document S1. Figures S1–S6 and Tables S1 and S2 [file mmc1.pdf]

iScience, Volume *24*

Supplemental information

DNA repair factor KAT5 prevents ischemic acute kidney injury through glomerular filtration regulation

Akihito Hishikawa, Kaori Hayashi, Akiko Kubo, Kazutoshi Miyashita, Akinori Hashiguchi, Kenichiro Kinouchi, Norifumi Yoshimoto, Ran Nakamichi, Riki Akashio, Erina Sugita, Tatsuhiko Azegami, Toshiaki Monkawa, Makoto Suematsu, and Hiroshi Itoh

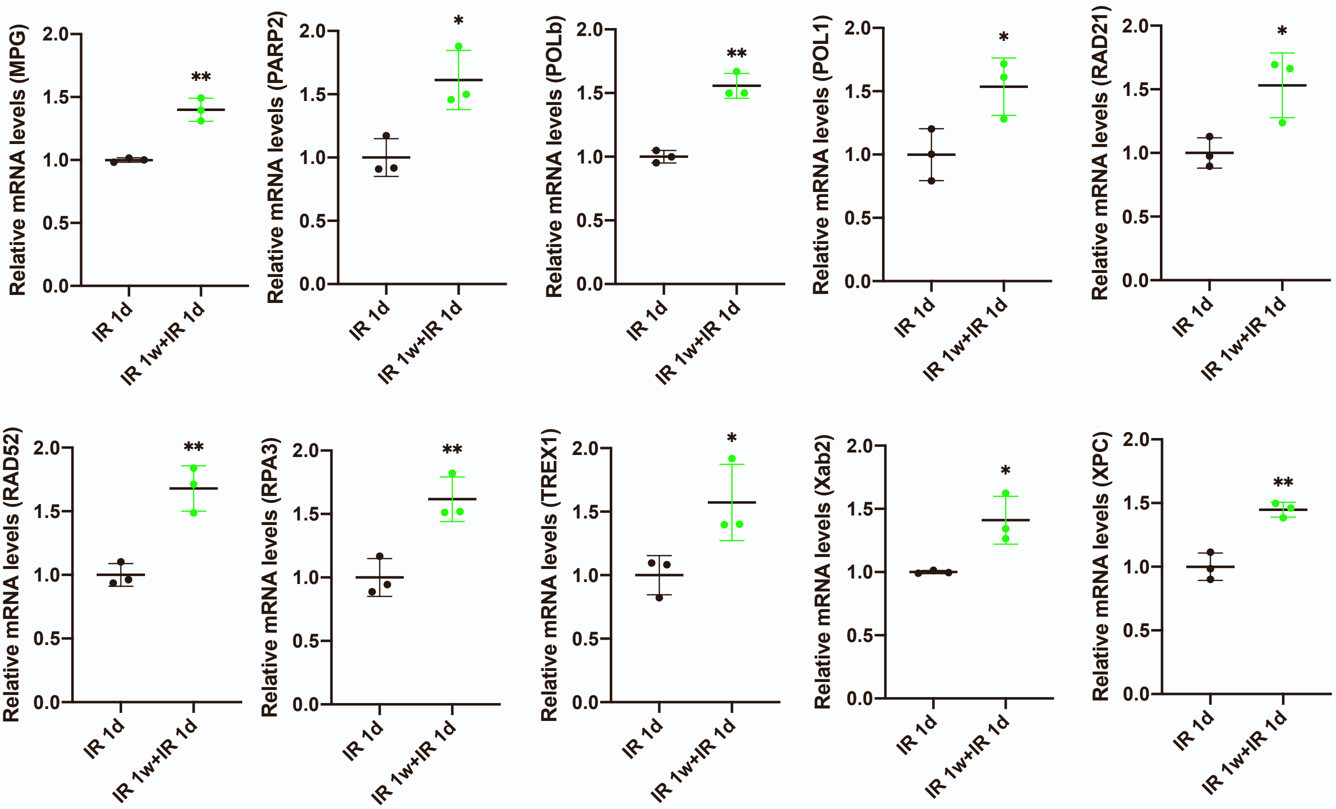

Supplementary Figure 1, related to Figure 1. Expression of DNA repair factors in the kidney cortex 24 hours after IR injury with or without preconditioning of 1 week prior to IR injury (IR1w+IR1d or IR1d, respectively), using PCR array. This figure indicates the factors with a significant increase in the preconditioning group compared with the IR group without preconditioning. The study protocol is indicated in **Figure 1A**. MPG: N-methylpurine-DNA-glycosylase, PAR: poly (ADP-ribose) , POL: DNA polymerase, TREX1:Three-prime repair exonuclease 1, Xab2: XPA-binding protein 2, XPC: xeroderma pigmentosum, complementation group C. Data represent the mean ± SEM. *: $p<0.05$, **: $p<0.01$ vs the respective groups.

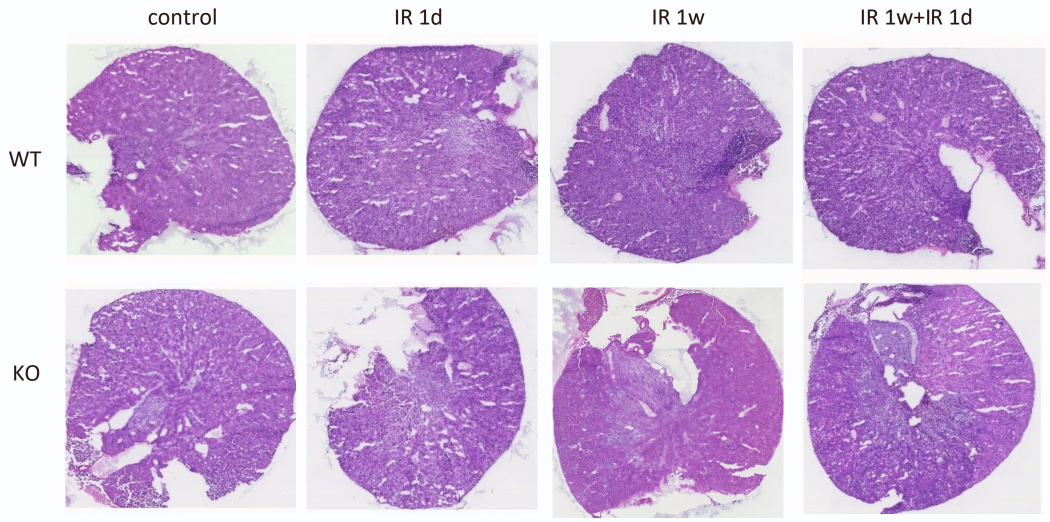

Supplementary Figure 2, related to Figure 4C. HE-stained images corresponding to **Figure 4C**.

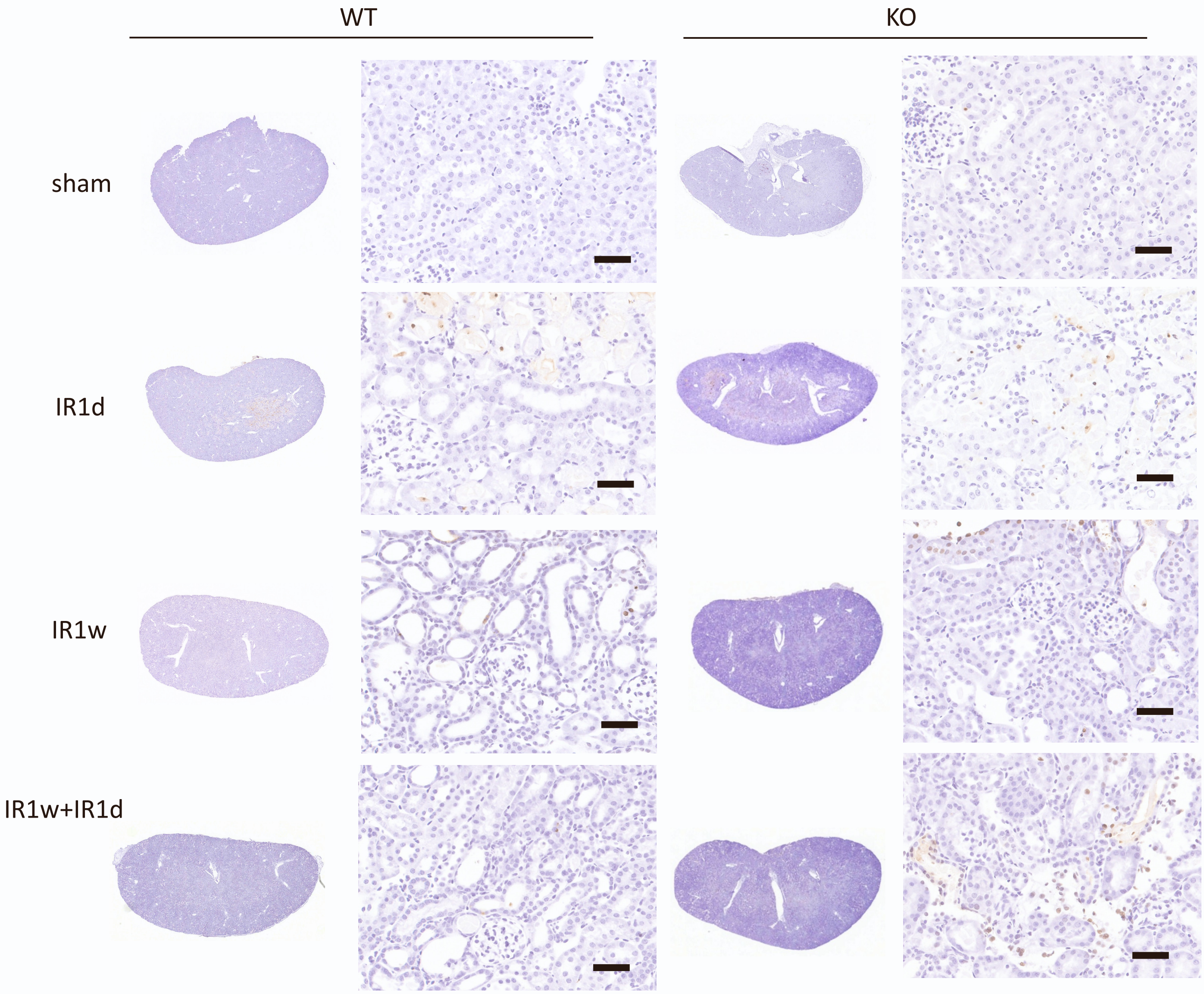

Supplementary Figure 3, related to Figure 4. Representative photomicrographs of TUNEL staining in the indicated groups as shown in **Figure 4**. Scale bar, 50 μm.

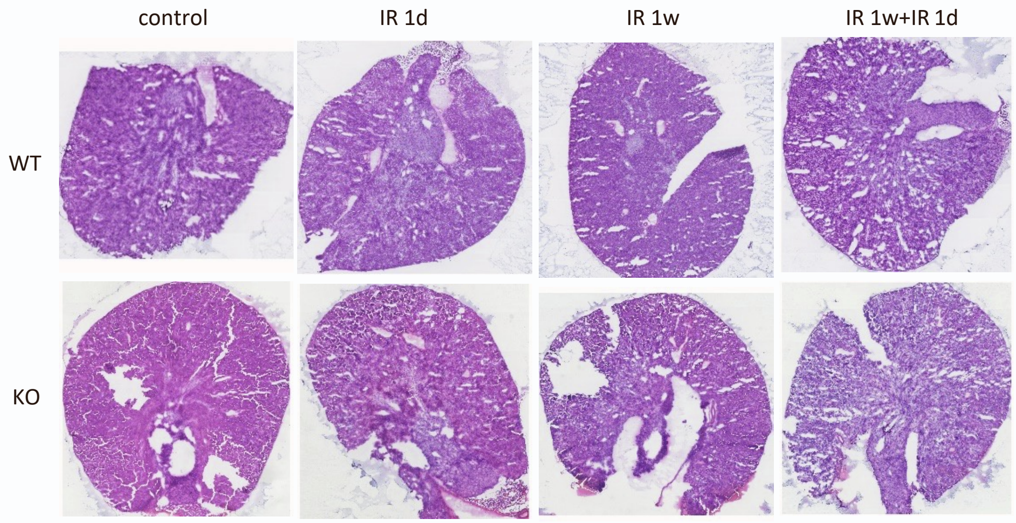

Supplementary Figure 4, related to Figure 5C. HE-stained images corresponding to **Figure 5C**.

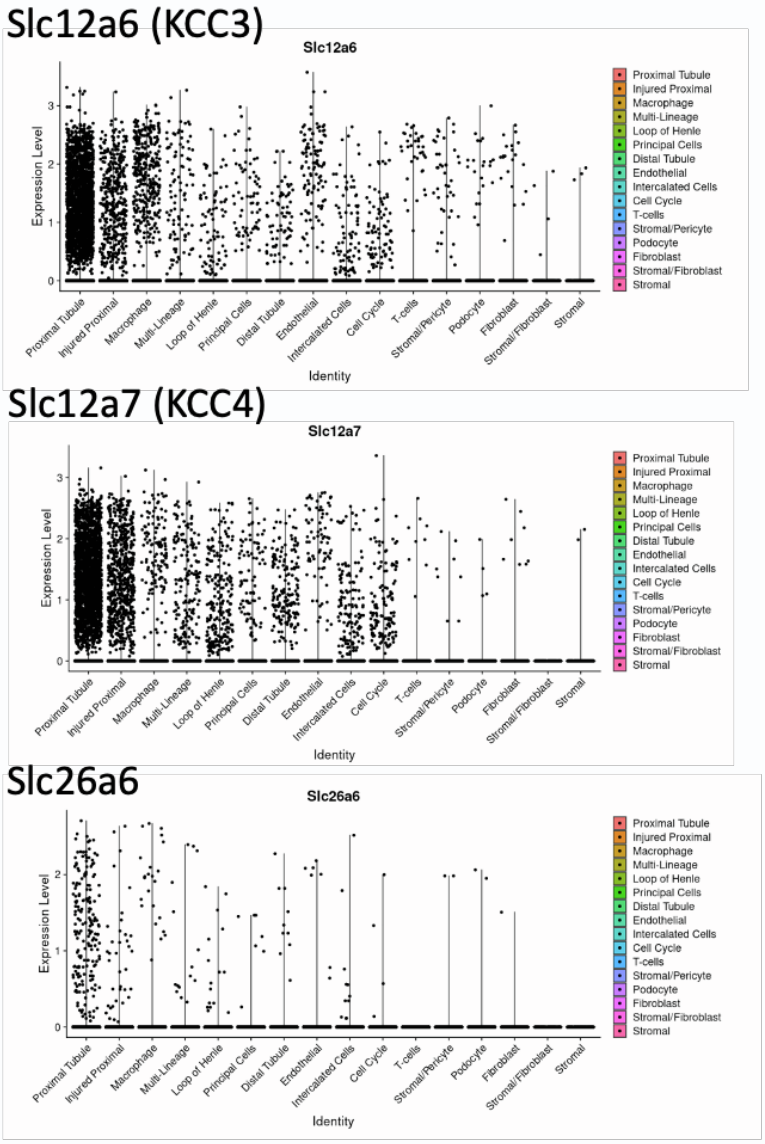

Supplementary Figure 5, related to Figure 6A. **Expression of KCC3, KCC4, and Slc26a6 using a database of a single-cell analysis reported previously.**

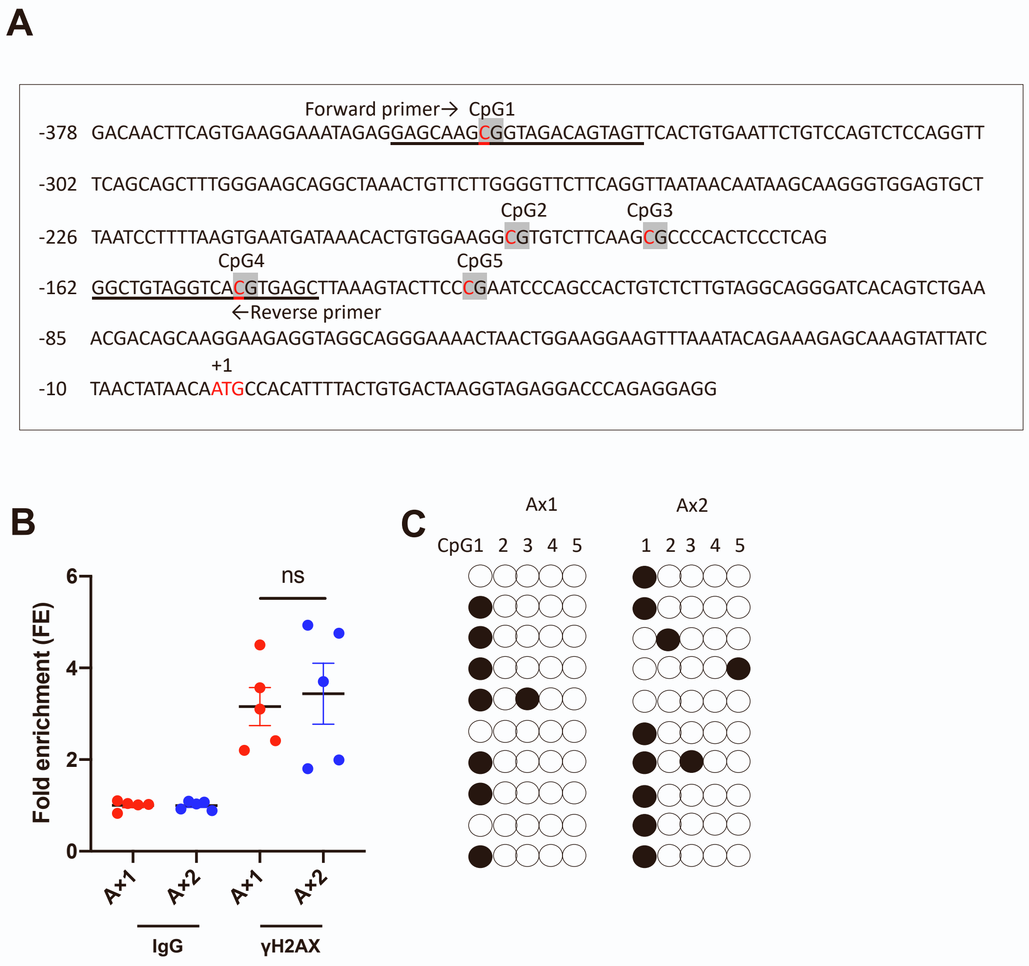

Supplementary Figure 6, related to Figure 7. (A) Maps of the KCC3 promoter region. Primers of chromatin accessibility assay and BGS and five CpG sites analyzed by BGS. (B) Quantitative ChIP assay performed using IgG and γH2AX antibodies. Data was normalized to input fraction and the results were relative to that of IgG which was set 1. (C) BGS analysis of the KCC3 promoter region in AMA-treated HK2 cells with or without pretreatment. The columns correspond to the CpG sequences shown in **Supplementary Figure 6A**. Each row represents a single sequenced clone. Empty dots: unmethylated CpGs; black dots: methylated CpGs. Data represent the mean ± SEM.

Supplementary Table 1, related to Figure 6H, I, J. Profiles of patients with kidney biopsy

	Non-AKI (n=11)	AKI (n=3)
Age (yr)	49±4.4	68±4.6
Sex (m/f)	4/7	2/1
eGFR (ml/min/1.73 m^2)	60±7.2	25.3±3.4*
proteinuria (g/gCr)	1.74±0.90	1.96±1.00
Diabetes	2	0

*p<0.05

Non-AKI and AKI groups include patients with minor glomerular abnormalities (MGA). AKI group includes patients diagnosed clinically and pathologically, with tubular injury recognized by skilled pathologists. AKI: acute kidney injury, eGFR: estimated glomerular filtration rate.

These data are obtained at the time of biopsy. Diabetes defined as either HbA1c>6.5% or prescription of diabetic medication.

Supplementary Table 2, related to STAR Methods. Sequence of oligonucleotide primers.

Primers for genotyping KAT5$^{flox/flox}$ mice

**Name**	**F/R**	**Sequence**
dPuro	F	GTCGCAGATACTCGGGAG
(WT: 198 bp, lox: 478 bp)	R	GGGAGTCTTAGCCAGGGC
dNeo	F	CCCAGTCACCAGGAGAGC
(WT: 227 bp, lox: 392 bp)	R	CTGCCCGCACAGCTCAGG

Real-time PCR primers

**Name**	**F/R**	**Sequence**
Mouse KAT5	F	GCCTGGACGGAAGCGGAAATCTAAT
	R	AAACACTTGGCCAGAAGACACAG
Mouse KIM-1	F	ATGAATCAGATTCAAGTCTTC
	R	TCTGGTTTGTGAGTCCATGTG
Mouse NGAL	F	CTCAGAACTTGATCCCTGCC
	R	TCCTTGAGGCCCAGAGACTT
Mouse KCC3	F	AGATGTCCTCAGTTCGGTTCA
	R	TCTGGCACACTTTCTCGGGA
Mouse KCC4	F	ATGCCCACGAACTTTACGGTG
	R	GGGAGGTTTGATCCACGCT
Mouse Slc26a6	F	GTGGCGAACTTGGTTCCGAT

	R	AGCCATTCACGCACAGGATAC
Mouse GAPDH	F	ATGGCCTTCCGTGTTCCTAC
	R	TGATGTCATCATACTTGGCAGG
Human KAT5	F	GGGGAGATAATCGAGGGCTG
	R	TCCAGACGTTTGTTGAAGTCAAT
Human KCC3	F	TCACAGTGATGACGCACTCAA
	R	CCAAGATGGACACAATGACACA
Human GAPDH	F	TGATGACATCAAGAAGGTGG
	R	TTTCTTACTCCTTGGAGGCC

BGS/ChIP/Chromatin accessibility primers

**Name**	**F/R**	**Target sequence**
Human KCC3 promoter BGS	F	GCTCACGTGACCTACAGCC
	R	GCTCACGTGACCTACAGCC

siRNA oligonucleotides

**Name**	**Sequence**
KAT5 stealth RNAi (NM_006388.2 #2)	CCGGATGAAGAACATTGAGTGCATT
